# Radiant Motherhood

**Published:** 1920-12-11

**Authors:** Marie Carmichael Stopes


*To the Editor of* THE HOSPITAL.

### "Radiant Motherhood."

*To the Editor of* THE HOSPITAL.

SIR,—Your reviewer, in attempted justification of his extraordinary phrase "grotesque errors," brings forward a number of points based on his own *opinions*, but not one solitary instance of a fully established, universally recognised, scientific fact about which I err.

A "grotesque error" it would have been had I asserted, for instance, that common salt is compounded of five atoms of sodium and one of chlorine, or had I asserted that the fœtal development takes place during eleven calendar months, or that the embryo develops actual fish gills during its process of metamorphosis. But not one instance of "error" cited by your reviewer is on this plane at all—all depend on his opinion.

No scientist of equal standing with my own has ever dreamed of applying the words "grotesque error" to any of my numerous published statements, discoveries, and views, and I cannot imagine any scientist of first-rate standing ever applying such a phrase to the views of another, whether he agrees with those views or not.

Unable to bring forward any instance of real error in my book, your reviewer makes the best he can of his position by submitting that my language is exaggerated when I state that with some women the abiding horror of childbirth is so great that they refuse to bear another child, and he professes to know only one case where it has been so. Your reviewer is probably the type of doctor of whom women write and speak to me, saying: "Of course I do not tell my doctor my reasons; I do not tell him anything; he is so unsympathetic and does not understand."

The next point raised by your reviewer is that the first sentence on page 57 is too absurd to be taken seriously. It is not absurd, it is tragically true; but again, objection to it is merely the opinion of your reviewer. When he says that I "hedge" about nausea in expectant mothers who are exceptional, he is again using an unworthy method of criticism, seeing that my book is specifically addressed to those who are healthy, happy, and normal, and explicitly puts out of consideration those who are diseased and are to be dealt with by doctors of disease. The further points also, all being arguable *opinions*, do not justify your reviewer's phrase either.

As the editor of a paper is always responsible for unsigned matter in his columns, I trust in the interests of THE HOSPITAL the Editor will tender me his apology for the unprincipled and unjustified use of the words "grotesque errors" concerning my book. If this is not done the status of the paper must seriously suffer in the eyes of all who are accustomed to the true methods of scientific controversy or criticism.—Yours faithfully,

